# Assessment of fourth‐year medical students' knowledge of post‐acute care rehabilitation discharge options following a physical medicine and rehabilitation clerkship educational program

**DOI:** 10.1002/pmrj.70222

**Published:** 2026-10-23

**Authors:** Elaine Zhou, Einat Engel‐Haber, Brittany Snider, Steven Kirshblum

**Affiliations:** ^1^ Department of Physical Medicine and Rehabilitation Rutgers New Jersey Medical School Newark New Jersey USA; ^2^ Kessler Foundation West Orange New Jersey USA; ^3^ Kessler Institute for Rehabilitation West Orange New Jersey USA

## Abstract

**Background:**

Post‐acute care (PAC) plays an increasingly critical role in the health care system due to an aging population, shorter hospital stays, and the complex needs of patients. However, studies show that medical students often lack essential knowledge of PAC options, which may compromise safe care transitions. In the United States, medical education provides limited training on PAC and the rehabilitation requirements associated with these settings.

**Objective:**

To assess fourth‐year medical students' knowledge of and confidence in PAC options following a targeted educational program delivered within a physical medicine and rehabilitation (PM&R) clerkship.

**Design:**

Pre‐ and postintervention surveys.

**Setting:**

A single institution U.S. allopathic medical school.

**Participants:**

Fourth‐year medical students enrolled in the mandatory PM&R clerkship between January and May 2025. Of 104 eligible students, 53 completed both surveys and were included in the analysis.

**Intervention:**

The mandatory 2‐week PM&R clerkship included a didactic lecture that (1) reviewed admission and rehabilitation eligibility criteria for each PAC setting based on Centers for Medicare and Medicaid Services guidelines; (2) highlighted differences in physician oversight, nursing care, and average length of stay; and (3) applied concepts through patient case scenarios.

**Main Outcome Measures:**

The change from medical students' baseline to postclerkship knowledge of and confidence in PAC options.

**Results:**

Students showed statistically significant improvements in both knowledge of PAC eligibility criteria (*p* < .001) and self‐reported confidence in PAC discharge planning (*p* < .001).

**Conclusions:**

A structured educational module on PAC in addition to clinical experiences in a mandatory PM&R clerkship was associated with improved medical students' knowledge and confidence in PAC discharge planning. These findings support incorporating PAC education into medical school curricula, particularly within PM&R rotations, to better prepare future physicians for the complexities of care transitions.

## BACKGROUND

The use of post‐acute care (PAC) settings is increasing due to the aging population and shorter duration of hospital stays.^1^, ^2^ Approximately 40% of Medicare beneficiaries discharged from acute‐care hospitals transition to PAC services.^3^ Patients discharged to PAC are often medically complex and face higher risks for hospital readmission.^4^, ^5^, ^6^ Effective communication with PAC facilities and proper education about PAC among healthcare providers is essential to reduce readmissions and promote safe discharge transitions.^7^, ^8^, ^9^, ^10^, ^11^, ^12^ With an increasing reliance on PAC, integrating comprehensive training on these options into medical education is crucial.

Medical students play vital roles within the healthcare team by helping patients navigate the healthcare system and facilitating transitions in patient care.^13^, ^14^ Medical education in the United States often prioritizes instruction in acute care settings and lacks adequate education in PAC rehabilitation.^15^, ^16^, ^17^, ^18^ Consequently, medical students often fail to receive adequate education in post‐acute rehabilitation and recovery following acute hospitalization for various conditions, including trauma, stroke, and numerous other acute and chronic debilitating illnesses.^15^, ^16^, ^17^, ^18^, ^19^ Trainees must be proficient in the different types of PAC and know when and how to apply each appropriately. Focused education on PAC can better equip future physicians to counsel patients and advocate effectively during transitions of care.

A recent study assessing the knowledge of medical students during their clinical rotations found that they lacked essential understanding of PAC settings and rehabilitation.^19^ Failure to provide such education to medical students can result in future residents and practicing physicians who lack the ability to safely transition patients to PAC. Notably, a recent cross‐sectional survey of internal medicine residents identified significant knowledge gaps regarding PAC and found discordance between residents' self‐assessed knowledge and their confidence, indicating inappropriately high confidence levels.^15^

PAC and rehabilitation education enables medical students to gain the skills necessary to provide anticipatory guidance when caring for acutely hospitalized patients with new or ongoing disabilities and rehabilitation needs during transitions to the appropriate care settings. Physical medicine and rehabilitation (PM&R) clerkships provide important exposure to the fundamental principles and practices of rehabilitation but are not typically mandatory in most United States medical schools.^20^, ^21^, ^22^ The aim of this study was to determine the knowledge of fourth‐year medical students on PAC options and the impact of implementing an educational program in the curriculum of a PM&R clerkship that specifically focuses on PAC rehabilitation options and the eligibility criteria for each.

## METHODS

### Study overview and design

Fourth‐year medical students from Rutgers New Jersey Medical School rotating through the mandatory PM&R clerkship were recruited to participate in the study. This study was approved by the local institutional research board and involved an anonymous pre‐post interventional survey using Research Electronic Data Capture. A preintervention questionnaire that assessed the baseline knowledge and confidence of medical students on PAC rehabilitation options and eligibility criteria was administered prior to the didactic series. Information was collected on prior experience in PM&R and prior formal education related to PAC. The questionnaire included six case scenarios in which participants were asked to identify the most appropriate PAC setting, as well as three multiple‐choice questions assessing knowledge of PAC setting requirements. Answers to these cases and questions were not provided at that time. Participants were also asked to rate their confidence level in their ability to determine the most suitable PAC setting.

During the PM&R clerkship, students were assigned to a primary clinical setting, which included inpatient services, consult services, or outpatient clinics. Although each student had a designated primary service, the clerkship was structured to provide exposure across multiple clinical environments. Students were encouraged to spend time in alternate settings during the rotation (eg, those primarily assigned to inpatient services may observe outpatient clinics, and vice versa) to broaden their exposure to different aspects of rehabilitation medicine and patient care transitions. After completion of the PM&R rotation, which included a 1‐hour lecture on PAC settings as the intervention, a postclerkship questionnaire with the same questions was administered. We used the first year of this program (in 2024) as a pilot study to refine the hypothetical cases and survey questions, and the findings presented in the subsequent sections pertain to the questionnaire administered in the second year of the program (in 2025). We designed this program as an introductory educational experience that provided targeted information and practical, actionable learning points related to PAC discharge planning.

### Participants

Fourth‐year medical students rotating through the PM&R clerkship between January and May 2025. The students received a $15 gift card upon completion of both (pre‐ and postclerkship) surveys.

### Didactic session (the intervention)

Students received a 1‐hour interactive lecture on PAC discharge options, during which eligibility criteria and admission requirements for each of the individual settings (ie, skilled nursing facility [SNF], acute inpatient rehabilitation facility [IRF], long‐term acute care facility [LTACH], and home with home care services) were explained in detail based upon Centers for Medicare and Medicaid (CMS) guidelines.^23^ In this study, we focused on CMS criteria because they are widely used in clinical practice and provide a standardized framework for understanding admission requirements for many PAC settings. The lecture incorporated patient case scenarios (different questions assessing the same concepts as the pre‐ and postclerkship surveys) and described the major medical, nursing, and therapy needs; intensity of treatment required; and prognosis for functional change for each case.

### Data analysis

The exact McNemar's test was used to determine statistically significant differences in the proportion of correct answers in the case scenarios and criteria questions. All analyses were considered statistically significant at *p* < .05. However, to address the issue of multiple comparisons, we applied the conservative Bonferroni correction approach. Consequently, for a result to be deemed significant after this correction, the adjusted *p* value was calculated to be *p* < .005. A paired *t*‐test was conducted to assess the improvement in scores.

A survey question on students' confidence in their ability to determine the most appropriate PAC plan was answered on a four‐point Likert‐style scale (very confident, somewhat confident, not very confident, not at all confident). A paired‐samples sign test was used to detect whether there was a median difference between the pre‐ and postsurvey responses for each participant.

## RESULTS

### Baseline characteristics

A total of 104 fourth‐year medical students were invited to participate in the study, with 64 completing the preintervention questionnaire and 53 completing both pre‐ and postintervention assessments. The analysis presented is based on the 53 participants with complete data sets. Of these participants, 55% identified as female and 45% as male. Twenty‐one percent had completed at least one prior PM&R rotation. The majority (91%) of students reported having had no prior formal education (lectures/workshops) related to PAC.

### Knowledge

As previously noted, student knowledge was assessed using six clinical vignettes and three direct multiple‐choice questions (see the Supporting Information document for a full description of the questions). For all nine questions combined, prior to the intervention, 56.8% (271/477) of responses were correct, with students averaging 5.1 ± 2.2 correct answers out of 9. Following the intervention, 83.4% (398/477) of responses were correct, with an average of 7.5 ± 2.3 correct answers. The improvement in mean scores was statistically significant (*p* < .001, paired *t*‐test) (Table 1).

**TABLE 1:** Pre‐ vs. postintervention correct responses on knowledge assessment questions (*N* = 53).

Question on	Preintervention	Postintervention	*p* value
Clinical vignette cases (*N* = 6)			
1. IRF	21 (39.6%)	43 (81.1%)	<.001
2. SNF	30 (56.6%)	40 (75.5%)	.078
3. Home care	46 (86.8%)	48 (90.6%)	.727
4. LTACH	33 (62.3%)	48 (90.6%)	<.001
5. SNF	31 (58.5%)	41 (77.4%)	.052
6. IRF	21 (39.6%)	47 (88.7%)	<.001
Total correct responses (out of 318)	**182 (57.2%)**	**267 (83.9%)**	**<.001**
Criteria questions (*N* = 3)			
1. SNF	21 (39.6%)	39 (73.6%)	.001
2. IRF	29 (54.7%)	45 (84.9%)	<.001
3. LTACH	39 (73.6%)	47 (88.7%)	.057
Total correct responses (out of 159)	**89 (56.0%)**	**131 (82.4%)**	**<.001**
All questions combined (*N* = 9)			
Total correct responses (out of 477)	**271 (56.8%)**	**398 (83.4%)**	**<.001**

*Note*: Data are presented as number of correct responses (%). Pre and post‐intervention responses were compared for individual questions and total correct responses.

Abbreviations: IRF, acute inpatient rehabilitation facility; LTACH, long‐term acute care facility; SNF, skilled nursing facility.

* *p* < .001.

When evaluating only the six vignettes to assess student knowledge of the most appropriate PAC placement for each scenario, the findings were similar to those reported in the preceding paragraph, with percentage of correct responses improving from 57.2% to 83.9% (*p* < .001).

An IRF was the most appropriate discharge placement for two cases presented, and correct responses on these cases significantly improved from 39.6% preintervention to 81.1% postintervention (*p* < .001) and from 39.6% to 88.7% (*p* < .001). For the case where LTACH was the most appropriate placement, correct responses improved significantly from 62.3% to 90.6% (*p* < .001). SNF was the most appropriate option for two cases, and correct responses improved from 56.6% to 75.5% (*p* = .078) and from 58.5% to 77.4% (*p* = .052), but these were not statistically significant. Responses for the case where home with in‐home or outpatient therapy was the most appropriate answer showed slight improvement from 86.8% to 90.6% (*p* = .727), but this was not statistically significant.

When the three questions to assess student knowledge of the different eligibility criteria were evaluated separately, students again demonstrated statistically significant improvements (*p* < .001) in their knowledge of eligibility criteria for various PAC settings following the educational intervention. The percentage of the correct responses closely mirrored those of the six clinical vignettes (56% pre and 82% post).

For SNF admission, the proportion of students correctly identifying the criterion of being able to participate in at least 1 hour of therapy per day increased from 39.6% to 73.6% (*p* = .001). Similarly, recognition of the need for participation in at least two therapy disciplines as a criterion for IRF placement rose from 54.7% to 84.9% (*p* < .001). For LTACH admission, the percentage of students correctly identifying medically complex needs as the key criterion slightly improved from 73.6% to 88.7%, but not significantly (*p* = .057).

### Confidence level

There was a statistically significant increase in students' self‐reported confidence in determining appropriate PAC discharge destinations. Prior to the intervention, 86.8% reported low confidence (“not at all” or “not very” confident). Postintervention, 100% of students reported feeling at least “somewhat confident,” with 28.3% reporting being “very confident” (*p* < .001) (Table 2).

**TABLE 2:** How confident are you in your ability to determine the most appropriate post‐acute care hospital discharge plan between the options of returning home, meeting criteria for acute inpatient rehabilitation, long‐term acute care hospitalization, and skilled nursing facility (subacute rehabilitation)?

	Preintervention (*N* = 53)	Postintervention (*N* = 53)
Very confident	0	15 (28.3%)
Somewhat confident	7 (13.2%)	38 (71.7%)
Not very confident	32 (60.4%)	0
Not at all confident	14 (26.4%)	0

*Note*: Data are presented as number of students (%). Pre‐ and post intervention confidence levels were compared using paired‐smaple signed test.

* *p* < .001.

### Feedback about educational program

After the program, the majority of students (73.6%) “strongly agreed” that the education received on PAC was helpful in guiding discharge planning. Lectures and case examples were rated most helpful (88.7%), followed by the rotation experience (54.7%). Students could select multiple options. Given that few students had previous exposure to PM&R before the clerkship, we were unable to analyze its possible impact (Appendix A).

## DISCUSSION

This study demonstrates that integrating a targeted educational intervention within a PM&R clerkship was associated with significantly improved medical students' knowledge of and confidence in PAC discharge planning in terms of available settings and eligibility criteria. By introducing these concepts during the PM&R clerkship, the program addresses a critical educational gap in medical school training that is often overlooked despite the increasing reliance on PAC services in our health care system.^15^, ^16^, ^17^, ^18^ The most current information available regarding PAC settings and rehabilitation admission criteria was used in developing the lecture, which will be periodically reviewed and updated to reflect current regulatory guidance, payer policies, and clinical practices for future cohorts.

A unique feature of our program is its combination of a didactic lecture and experiential learning during the clinical rotation. This multimodal approach allowed students to contextualize complex discharge planning decisions and understand the eligibility criteria that guide transitions to SNFs, LTACHs, and IRFs, as well as to home. Our findings build on previous studies that identified widespread knowledge gaps in this area, demonstrating that even brief, focused interventions can result in meaningful short‐term improvements in both knowledge and confidence levels.^20^, ^21^, ^22^ The parallel improvements in both self‐assessed confidence and objective knowledge strongly suggest that students were better equipped to navigate discharge decisions postintervention.

Overall, students demonstrated consistent improvements in knowledge across all clinical scenarios. These gains suggest increased familiarity with the factors that influence PAC decision‐making, including functional status, medical complexity, and therapy tolerance. Although the extent of improvement varied between cases, the upward trend across all scenarios highlights the value of targeted education in conjunction with clinical experiences in strengthening students' understanding of PAC transitions. Nonetheless, further education in these areas is still needed.

A lack of knowledge among future physicians regarding appropriate PAC placements for medically complex patients  may potentially contribute to poorer health outcomes and avoidable readmissions.^9^, ^10^, ^11^, ^12^ Importantly, this educational intervention has relevance beyond medical students pursuing PM&R. The majority of participants in this study were fourth‐year medical students entering a wide range of specialties. Regardless of specialty, physicians are frequently involved in determining PAC needs or counseling patients and families on rehabilitation options. As the healthcare system continues to emphasize value‐based care, it is critical that physicians in training, including medical students and residents, are competent in PAC discharge planning.

Our program provides a replicable model for other institutions seeking to integrate PAC content into their curricula, particularly in settings where PM&R exposure may otherwise be limited. Importantly, students overwhelmingly found the educational content helpful and relevant, with many noting the dual value of the didactic lecture and experiential learning. These preferences support continued incorporation of multimodal education strategies in future iterations of the curriculum. Although this study evaluated these concepts within the context of a PM&R clerkship, the didactic component may be adaptable to other areas of the medical school curriculum. For example, a focused educational session on PAC decision‐making could be incorporated into clinical rotations, such as internal medicine, where discharge planning and transitions of care are integral to daily practice. Additionally, introducing this content within early clinical or health systems science curricula may provide medical students with an early framework for understanding transitions from acute care to rehabilitation and other postacute settings.

## STUDY LIMITATIONS

This study has limitations that should be considered when interpreting the results. First, the participant sample was drawn from a single institution, which may limit the generalizability of the findings to other medical schools with different curricula, clinical exposure, or student populations. The study had a completion rate of ~50% of the total sample population, which may limit the statistical power for detecting smaller effect sizes, particularly in questions that trended toward significance but did not reach conventional thresholds after correction for multiple comparisons. Potential selection bias cannot be ruled out, as those who completed both surveys may have been more motivated to learn from the clerkship.

An additional limitation is the variability in students' clinical experiences during the PM&R rotation, which may have influenced their ability to recognize appropriate PAC placements. Although all students received the same didactic education, their exposure to discharge planning and PAC decision‐making likely varied depending on their clinical setting. Students assigned to inpatient rehabilitation or hospital consult services may have had more direct involvement in assessing functional status and participating in care transition discussions than those placed in outpatient settings. This variability in clinical exposure could have contributed to differences in individual performance on the postintervention assessment. Nonetheless, this would only limit the extent of the improvement and would not minimize it.

The absence of a control group that completed the clerkship without the educational intervention limits our ability to attribute knowledge gains solely to the didactic session. Opportunities for structured discussion of specific PAC discharge placement decisions may vary depending on the clinical rotation and setting. Therefore, the lecture was implemented to ensure that all students received standardized education on postacute care options and eligibility criteria. Although some improvements in knowledge may have been influenced by clinical exposure during the clerkship, the didactic session was designed to address key concepts that may not consistently arise during routine clinical experiences. Nevertheless, further investigation is needed to assess the effectiveness of the didactic lecture independent of the clerkship experience and to clarify the individual contributions of the lecture and clinical exposure to the observed improvements in student knowledge.

Lastly, this study assessed knowledge and confidence immediately following the intervention but did not evaluate the long‐term retention of knowledge or the translation of learning into clinical practice. Future studies with longitudinal follow‐up would be needed to assess the durability and practical impact of the educational gains to determine whether knowledge gains persist into residency and translate into clinical decision‐making practices.

## CONCLUSION

The integration of a structured educational module consisting of didactic lectures on PAC in addition to clinical experiences within a mandatory PM&R clerkship was associated with significantly improved confidence and knowledge in PAC discharge planning among fourth‐year medical students. These findings support the inclusion of PAC education in medical school curricula, particularly within PM&R rotations, to better prepare future physicians for the complexities of care transitions.

## FUNDING INFORMATION

No funding was used in this study. No financial benefits to authors. A poster presentation was given at the Annual Physiatry 2025 conference.

## DISCLOSURE

There are no known conflicts of interest.

## Supporting information


**Data S1:** Supporting Information.

## Data Availability

The data that support the findings of this study are available from the corresponding author upon reasonable request.
