# Supplementary material for: Assessment of fourth‐year medical students' knowledge of post‐acute care rehabilitation discharge options following a physical medicine and rehabilitation clerkship educational program
Source: PM R. 2026 Oct 23;18(11):1334–40. doi: 10.1002/pmrj.70222 (PMC13768719; doi:10.1002/pmrj.70222)
Supplement: Supplementary file 1 [file PMRJ-18-1334-s001.docx]

**Clinical Case Vignettes**

Correct answers are bolded

**Case 1:**

A 72 year old male with history of hypertension and atrial fibrillation fell at home and sustained a subdural hematoma. He is awake, moving all extremities, intermittently confused and impulsive. He has started physical therapy and is able to walk 30' with moderate assistance and felt to be able to tolerate multiple hours of therapy interventions. He is married and lives in a home with 10 stairs to enter. Prior function - independent with ambulation and activities of daily living (ADLs). Family and patient are requesting referral for further rehabilitation. What is the most appropriate discharge destination for this patient?

| **Answer Choice** |  |
| --- | --- |
| **A** | **Acute inpatient rehabilitation** |
| B | Home with in-home or outpatient therapy |
| C | Long term acute care hospital (LTACH) |
| D | Skilled nursing facility (SNF) |

**Case 2:**

88 year old male with history of mild dementia and recent total hip replacement is being treated for infection and requires 6 weeks of IV antibiotics. He is moving all extremities, awake, intermittently confused and impulsive. **He has started physical therapy and is able to stand and walk 5' with maximum assistance, and it was felt that he would not be able to tolerate multiple hours of therapy interventions.** He is married and lives in a home with 10 stairs to enter. Prior function - modified independent for ambulation with a walker. Family is requesting referral for further rehabilitation. What is the most appropriate discharge destination for this patient?

| **Answer Choice** |  |
| --- | --- |
| A | Acute inpatient rehabilitation |
| B | Home with in-home or outpatient therapy |
| C | Long term acute care hospital (LTACH) |
| **D** | **Skilled nursing facility (SNF)** |
| E | Other |

**Case 3:**

42 year-old female with no past medical history trips while descending stairs at a concert. She presents to the ER with complaints of right ankle pain and swelling. Imaging is negative for fracture. She is evaluated by orthopedic surgery who diagnoses a right ankle sprain. She is prescribed a walking boot for the right foot and is cleared for full weight-bearing with the use of the boot. **She has started physical therapy and can walk up to 150 feet with a cane**. She lives with a roommate in an elevator-accessible apartment with no steps to enter. OT documents the patient is independent with activities of daily living (ADLs) although she requires increased time to complete tasks. The patient is very concerned about the pain. Patient is requesting referral for further rehabilitation. What is the most appropriate discharge destination for this patient?

| **Answer Choice** |  |
| --- | --- |
| A | Acute inpatient rehabilitation |
| **B** | **Home with in-home or outpatient therapy** |
| C | Long term acute care hospital (LTACH) |
| D | Skilled nursing facility (SNF) |
| E | Other |

**Case 4:**

64 year-old man had cardiac arrest while at the gym. Automated external defibrillator was used and the patient had return of spontaneous circulation within 4 minutes. He was taken to cath lab on admission for attempted revascularization. The patient arrested again during the procedure. He subsequently underwent 3 vessel CABG. Post-operatively he developed ARDS requiring prolonged mechanical ventilation. A tracheostomy and PEG tube were placed. He is now 14 days from admission and is still on the ventilator, however he requires intermittent continuous venovenous hemodiafiltration due to hypoperfusion injury to the kidneys. He remains in the CCU and is making slow, steady progress with weaning. He is very deconditioned, **not able to participate in lengthy therapy at this time,** but tolerating sitting in bedside chair for up to 1 hour. He is awake and alert, but responses are delayed. Family is requesting referral for further rehabilitation. What is the most appropriate discharge destination for this patient?

| **Answer Choice** |  |
| --- | --- |
| A | Acute inpatient rehabilitation |
| B | Home with in-home or outpatient therapy |
| **C** | **Long term acute care hospital (LTACH)** |
| D | Skilled nursing facility (SNF) |

**Case 5**:

75 year-old woman presents to ED with fever of 102.10 F, shortness of breath and cough for the last 3 days. She tests positive for COVID. Chest x-ray is negative for pneumonia and she does not require supplemental oxygen. She has very limited activity tolerance and requires assistance for transfers, toileting and short distance ambulation. She is admitted to the Observation Unit and started on intravenous fluids and Tylenol. On hospital day # 10**, her fever has resolved and medical status has improved except her fatigue level. She has started therapy and is able to stand and walk < 10' with significant fatigue, and felt that she would not be able to tolerate multiple hours of therapy interventions.** She lives alone in ranch-style home with 3 steps to enter. She previously had limited mobility to household ambulation. Family is requesting referral for further rehabilitation. What is the most appropriate discharge destination for this patient?

| **Answer Choice** |  |
| --- | --- |
| A | Acute inpatient rehabilitation |
| B | Home with in-home or outpatient therapy |
| C | Long term acute care hospital (LTACH) |
| **D** | **Skilled nursing facility (SNF)** |
| E | Other |

**Case 6**:

76 year-old man suffered a stroke with residual right hemiparesis 3 days earlier. He has a history of diabetes mellitus type 2 and hypertension. He lives alone in ranch-style home with 4 steps to enter. **He has started physical therapy and is able to walk 25' with moderate assistance, has limitations in feeding himself and has fluent aphasia**. It is felt that he is able to tolerate multiple hours of therapy interventions He was independent with ambulation and activities of daily living (ADLs) prior to this hospitalization. Patient is requesting referral for further rehabilitation. What is the most appropriate discharge destination for this patient?

| **Answer Choice** |  |
| --- | --- |
| **A** | **Acute inpatient rehabilitation** |
| B | Home with in-home or outpatient therapy |
| C | Long term acute care hospital (LTACH) |
| D | Skilled nursing facility (SNF) |
| E | Other |

**Criteria Questions**

**1. Criteria for admission to a skilled nursing facility/subacute rehabilitation includes:**

| **Answer Choice** |  | Pre-intervention | Post-intervention |
| --- | --- | --- | --- |
| **A** | 15 hrs therapy per week | 15 (28.3%) | 2 (3.8%) |
| B | Daily physician visits | 3 (5.7%) | 0 |
| **C** | **<1 hr daily therapy** | **21 (39.6%)** | **39 (73.6%)*** |
| D | >= 2 therapy disciplines | 9 (17%) | 4 (7.5%) |
| E | None | 5 (9.4%) | 8 (15.1%) |

*p=0.001

**2. Which of the following is a criterion for admission to an acute rehabilitation facility:**

| **Answer Choice** |  | Pre-intervention | Post-intervention |
| --- | --- | --- | --- |
| A | Not require any medical/nursing services | 6 (11.3%) | 2 (3.8%) |
| B | Able to participate in less than 1 hour of therapy per day | 10 (18.9%) | 2 (3.8%) |
| C | Be on a ventilator | 3 (5.7%) | 0 |
| **D** | **Requires at least two therapy disciplines (e.g., OT, PT, SLP)** | **29 (54.7%)** | **45 (84.9%)*** |
| E | None | 5 (9.4%) | 4 (7.5%) |

*p<0.001

**3. Which of the following is a criterion for admission to a long term acute care (LTACH) facility:**

| **Answer Choice** |  | Pre-intervention | Post-intervention |
| --- | --- | --- | --- |
| **A** | **Medically complex needs (e.g. ventilator weaning)** | **39 (73.6%)** | **47 (88.7%)*** |
| B | Limited physician visits required to two times per week | 4 (7.5%) | 0 |
| C | Must not have been in the intensive care unit (ICU) | 4 (7.5%) | 2 (3.8%) |
| D | Requires at least two therapy disciplines (e.g., OT, PT, SLP) | 1 (1.9%) | 2 (3.8%) |
| E | None of the above | 5 (9.4%) | 2 (3.8%) |

* p=0.057
